# Distinct functional defect of three novel Brugada syndrome related cardiac sodium channel mutations

**DOI:** 10.1186/1423-0127-16-23

**Published:** 2009-02-20

**Authors:** Chia-Hsiang Hsueh, Wen-Pin Chen, Jiunn-Lee Lin, Chia-Ti Tsai, Yen-Bin Liu, Jyh-Ming Juang, Hsuan-Ming Tsao, Ming-Jai Su, Ling-Ping Lai

**Affiliations:** 1 Institute of Pharmacology, School of Medicine, National Taiwan University, Taipei, Taiwan; 2 Department of Internal Medicine, National Taiwan University Hospital, Taipei, Taiwan; 3 Department of Internal Medicine, I-Lan Hopital, I-Lan, Taiwan

## Abstract

The Brugada syndrome is characterized by ST segment elevation in the right precodial leads V1-V3 on surface ECG accompanied by episodes of ventricular fibrillation causing syncope or even sudden death. The molecular and cellular mechanisms that lead to Brugada syndrome are not yet completely understood. However, SCN5A is the most well known responsible gene that causes Brugada syndrome. Until now, more than a hundred mutations in SCN5A responsible for Brugada syndrome have been described. Functional studies of some of the mutations have been performed and show that a reduction of human cardiac sodium current accounts for the pathogenesis of Brugada syndrome. Here we reported three novel SCN5A mutations identified in patients with Brugada syndrome in Taiwan (p.I848fs, p.R965C, and p.1876insM). Their electrophysiological properties were altered by patch clamp analysis. The p.I848fs mutant generated no sodium current. The p.R965C and p.1876insM mutants produced channels with steady state inactivation shifted to a more negative potential (9.4 mV and 8.5 mV respectively), and slower recovery from inactivation. Besides, the steady state activation of p.1876insM was altered and was shifted to a more positive potential (7.69 mV). In conclusion, the SCN5A channel defect related to Brugada syndrome might be diverse but all resulted in a decrease of sodium current.

## Background

SCN5A encodes the alpha subunit of human cardiac sodium channel, which is responsible for the generation of cardiac action potential and for rapid impulse conduction through the myocardium[1]. Mutations in SCN5A cause inherited arrthymia syndrome such as Long QT syndrome (LQT3), Brugada syndrome, isolated conduction disease, atrial stanstill, congenital sick sinus syndrome or sudden infant death syndrome [2,3]. Chen et al. first reported in 1998 that loss of function mutations of SCN5A accounts for the most well-known genetic basis for Brugada syndrom[4]. However, for the clinically diagnosed cases, only no more than 20% carry SCN5A mutations[5]. Mutations in other genes that cause Brugada syndrome have been reported. These genes include glycerol-3-phosphate dehydrogenase 1-like gene (GPD1L), the alpha subunit of the L-type calcium channel (CACNA1C), the beta subunit of the L-type calcium channel (CACNB2b), and the sodium channel beta subunit (SCN1B) [6-8]. However, SCN5A is so far still the most often reported gene causing Brugada syndrome. Altered electrophysiology, trafficking, expression level, or interaction with its intracellular components all has been reported to account for the mechanisms contribute to loss of function of SCN5A [9-12]. In this study, we investigated on three SCN5A mutation identified in patients with Brugada syndrome in Taiwan and tried to identify the underlying mechanism of three mutations that contribute to Brugada syndrome.

## Materials and methods

### Cloning of SCN5A and SCN1B

Total RNA was extracted from human heart tissue using trizol (Invitrogen, USA) according to manufacturer's protocol. Complimentary DNA was synthesized using 200 units of Superscript III reverse transcriptase (Invitrogen, USA) at 52°C for 50 min in the presence of 5 μg of total RNA, 0.5 μg of oligo-dT primers, 0.004 mM DTT, 5% DMSO and 0.2 mM dNTPs, and the reaction product was used as the template in subsequent polymerase chain reaction(PCR). The PCR product for SCN1B was first cloned into pAAV-IRES-hrGFP (Stratagene, USA) with BamH I/Xho I and subcloned into the Bgl II recognition sequence of pBudCE4.1 (Invitrogen) with BamH I/Bgl II. The subclone procedure allowed dicistronic expression of SCN1B and humanized *Renilla reniformis *green fluorescent protein (hrGFP) under the control of EF-1α promoter. The PCR product of SCN5A was cloned into the pBudCE4.1 containing SCN1B with Hind III/Xba I under the control of CMV promoter. Besides, a myc epitope EQKLISEEDL was introduced in frame at the N terminus of SCN5A. The base sequence of the SCN5A and SCN1B clone were analyzed and were identical to the published SCN5A (hH1, NM_198056) and SCN1B (NM_199037) sequence.

### Site directed mutagenesis

The p.I848fs, p.R965C, and p.1876insM mutants were generated using the QuickChange Site-Directed Mutagenesis system (Stratagene). All constructs were sequenced to verify the mutation and to rule out possible PCR errors.

### Culture and transfection of HEK293T cells

HEK293T cells were grown in Dulbecco's modified Eagle's medium supplemented with 10% fetal bovine serum and antibiotics at 37°C and 5% CO2. Jetpei (Polyplus) was used as the reagent for transient transfection. Briefly, 3 × 10^5 ^HEK293T cells were seeded into a 35 mm dish the day before transfection. Onto the cell monolayer were added 3 μg plasmid and 6 μl Jetpei in 200 μgl sodium chloride solution (150 mM). The cells were harvested by PBS containing 1% EDTA for patch clamp or for western blot 48–56 hrs after transfection.

### Patch clamp and data analysis

Cells were incubated in bath solution containing (mM): NaCl 145, KCl 4, CaCl 1.8, MgCl 1, HEPES-NaOH pH 7.35. Expressed currents were recorded by whole-cell patch-clamp technique using a patch-clamp amplifier (Dagan 8900). The pipette solution contained (mM) NaF 10, CsF 110, CsCl 20, EGTA 10 and HEPES 10 (pH 7.35 with CsOH). Pipettes were made from borosilicate glass capillaries and had tip resistances between 1.5 and 2.5 MÙ when filled with the pipette solution. All of the electrical recordings were performed at room temperature (24–26°C). Data acquisition was performed through a DigiData 1200 amplifier controlled by pClamp 6.0, and the results were analyzed using Clampfit 9.0.

To measure the cell capacitance, a step voltage (from -80 mV to -70 mV) was applied to the cell and accessed the area under the capacity transient. The cell capacitance was obtained by dividing the area under the capacity transient with the factor 10. The plots of voltage dependent steady state activation and inactivation were fitted by Boltzmann equation: Y = 1/[1+exp(V -Vm)/k], where Vm is the voltage at which sodium current is half-maximally activated, and k was the slope factor. Time constants of inactivation were obtained by fitting the decaying phase of current trace with one exponential equation: Y = A*exp(-t/*τ*) + C. To analyze the kinetics of recovery from inactivation, two-exponential equation was as in the format: Y = A*[1-exp(-t/*τ*f)]+(1-A)*[1-exp(-t/*τ*s)].

### Immunocytochemistry and Confocal imaging

HEK293T cells were cultivated on chamber slips (UNUC) and transiently transfected with WT, p.I848fs, p.R965C, or p.1876insM. Forty-eight hours after transfection, the culture medium were removed and cells were washed with ice-cold PBS. The cells were then fixed by incubation in 4% paraformaldehyde at room temperature for 30 min and permeabilized with PBS containing 0.5% tween 20 and 10% BSA for another 30 min. Before comfocal imaging, cells were incubated in PBS containing primary antibody (anti-myc 1:500, Upstate) overnight at 4°C and secondary antibody anti-mouse IgG coujugated with Cy3 (1:2000, Sigma) for 30 min. Confocal images were obtained and analyzed using a Leica TCS SP5 Spectral Confocal System.

### Surface biotinylation reaction

Sulfo-NHS-LC-Biotin (PIERCE) was used as the reagent for labeling of cell surface proteins. Briefly, the transfected cells were washed and harvested using 1%EDTA (prepared in PBS). The cell pellets were resuspended in PBS containing Sulfo-NHS-LC-Biotin (2 mg/ml) and rotated at 4°C for 30 min. The biotinylation reaction was blocked by adding equal volume of 0.1 M glycine (in PBS) and rotated for mixing for 20 min at 4°C. The cells were then centrifuged and wash 3 times with PBS and subjected to protein extraction and Western blotting.

### Western blotting

The cells for protein extraction were collected 48 to 56 hours after transfection by centrifuging at 500 g for 5 min. The pellet was then incubated in lysis buffer (1% Triton-X 100 pH 8.0, 50 mM Tris-HCl, 300 mM NaCl, 5 mM EDTA, 0.02% sodium azide, 1 mM PMSF, 2 μg/ml leupeptin, and 5 μg/ml apotinin) for 30 min, and centrifuged at 15000 g for 15 min.

For analysis of biotinylated protein, the extracted proteins were mixed with anti-myc antibody (Upstate) and protein A/G plus (Santa Cruz) for 4 hrs, washed 4 times with lysis buffer and the immunoprecipitated proteins were released from the beads by heating at 95°C for 5 min in 5× sample buffer. The collected proteins were subjected to SDS-PAGE, transferred to PVDF membrane, and detected by Western blotting analysis using HRP conjugated streptavidin (PIERCE).

### Data management and statistical analysis

Data of patch clamp were ananlyzed using clampfit 9.0, and photoshop 8.0 was used for confocal image processing. Prism 4.0 was used for figure plotting, curve fitting and statistical calculation. Data were presented as mean ± standard error of the mean (SEM). Statistical comparisons were compared to WT using unpaired Student's t test for current density, Vm, slop factor, and time constants. Multiple group comparison was made using one way ANOVA followed by Tukey test. P values less than 0.05 were taken as statistically significant.

## Results

### Genetic analysis and the electrocardiographic (ECG) characteristics

**Figure 1:**
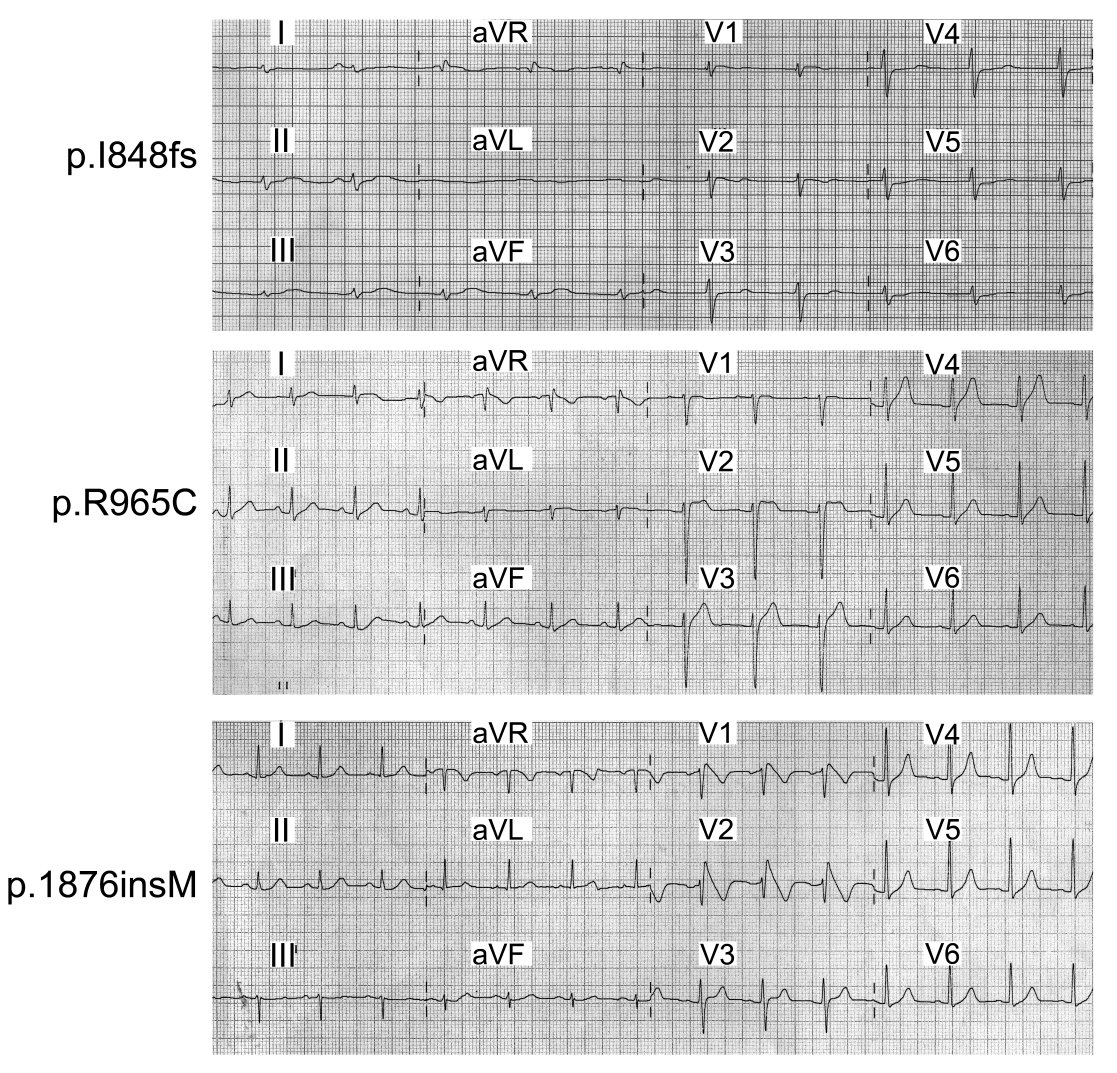
**Surface 12-lead ECG of the three patients with Brugada syndrome carrying SCN5A mutation**.

We had identified three SCN5A mutations in three patients with Brugada syndromes in Taiwan. Among them, two were novel (c.2540 del C, and c.5626 ins ATG) and one had been reported (c.2893 C>T)[13]. c.2540 del C introduced a new stop codon and thus produced a truncated protein with 880 amino acid residues (p.I848SfsX33). c.2893 C>T was a mutation in substitution form and changed the amino acid at position 965 of SCN5A form arginine to cysteine (p.R965C). The last mutation, c.5626 ins ATG had a 3 bp insertion and allowed an in frame insertion of methionine at amino acid 1876 (p.1876 insM). The surface 12-lead ECG of the three patients with Brugada syndrome carrying SCN5A mutation all revealed ST segment elevation characteristic of Brugada syndrome (Figure 1). The PR intervals for p.I848fs, p.R965C and p.1876insM carriers were 177, 180, and 174 ms respectively. They all had a similar clinical presentation of aborted sudden cardiac death due to ventricular fibrillation.

### Sodium current elicited by WT and mutant cardiac sodium channels

**Figure 2:**
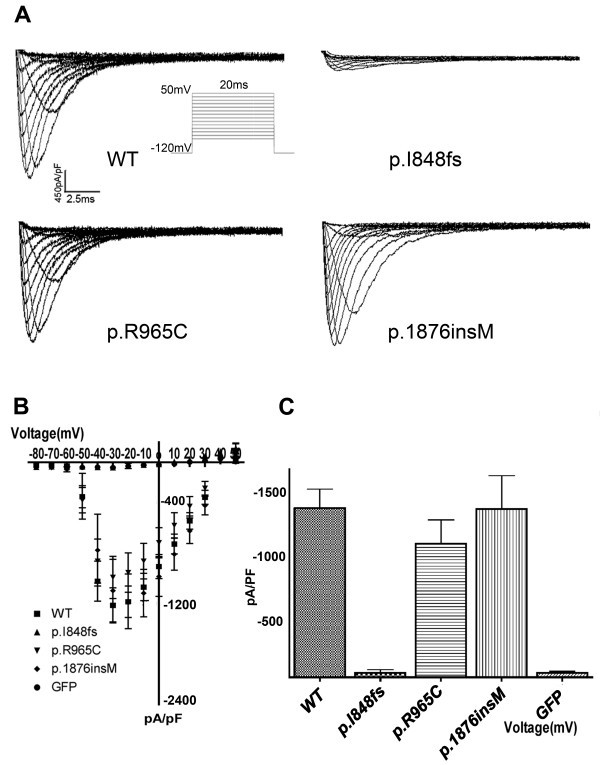
**Sodium current recorded on HEK293T cells transfected with SCN5A WT, or mutant plasmids**. (A) Representative current traces. Protocol used was shown in inset. (B) Current-voltage (I-V) relationship of WT, p.I848fs, p.R965C, and p.1876insM SCN5A channels (n = 9, 7, 7 and 11 respectively). GFP was used as a negative control and was also shown (n = 4). (C) Histogram of current density recorded at -20 mV. The current densities between WT and p.R965C or WT and p.1876insM were not statistically significance.

Figure 2A displayed the representative current traces of WT and three mutants. Their current-voltage relationships were shown in Figure 2B. WT, p.R965C, and p.1876 insM produced comparable sodium currents while p.I848fs elicited currents that were hardly detectable. The current densities among WT, p.R965C, or p.1876insM, were not statistically significant (Figure 2C).

### Voltage dependent steady state activation and steady state inactivation

**Figure 3:**
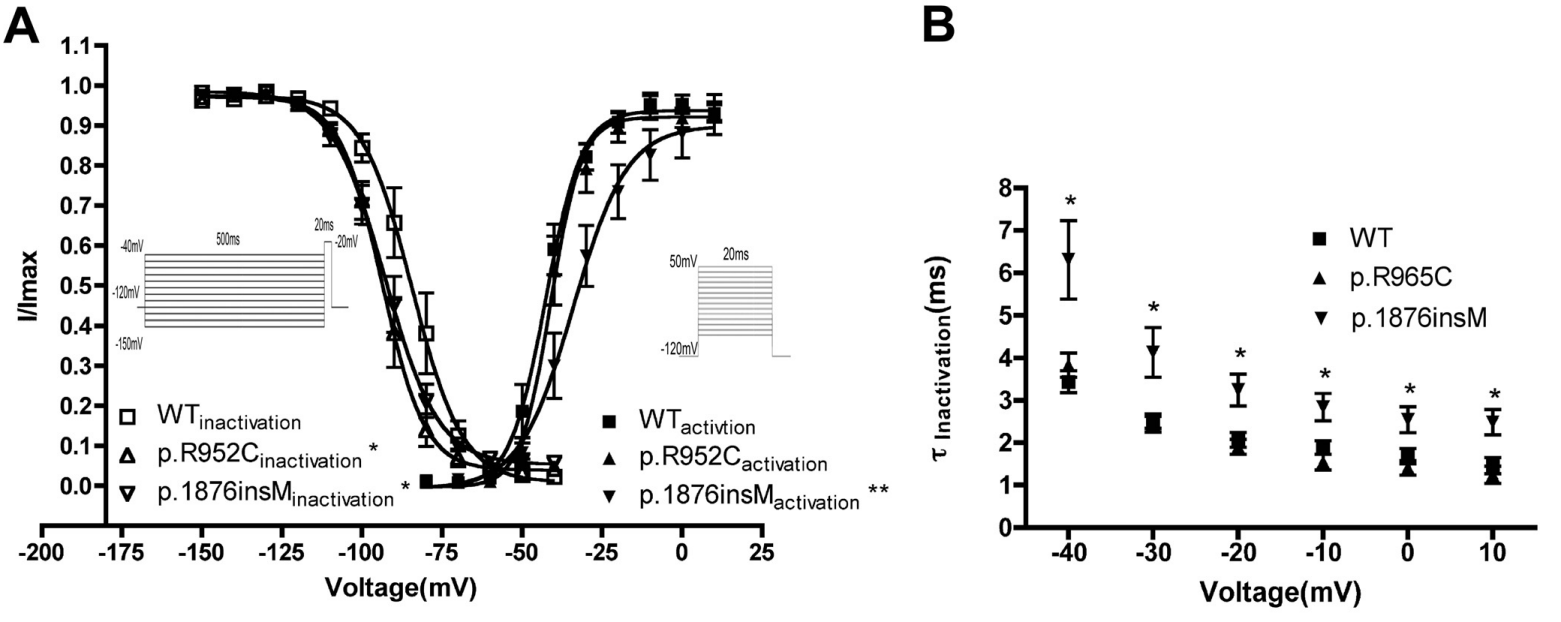
**Kinetics of sodium channel activation and inactivation**. (A) Voltage dependant steady state activation (n = 10,11, and 8 for WT, p.R965C, and p.1876insM respectively) and steady state inactivation (n = 10,11, and 8 for WT, p.R965C, and p.1876insM respectively). *, p < 0.05; **, p < 0.01 when compared to WT using one way ANOVA followed by Tukey test (B) Time constant of inactivation. This was accessed by fitting the decaying phase of current trace obtained as in Figure 2A with one exponential equation: Y = Aexp(-t/*τ*) + C. *, p < 0.05 when compared to WT

Since p.I848fs channels produced hardly detectable sodium current, we performed kinetics analysis for WT, p.R965C, and p.1876insM channels only. As shown in Figure 3A, the activation curves of WT and p.R965C were nearly superimposed. However, p.1876insM showed a right-shift of the steady state activation curve, indicating it required more a positive potential to open. When fitted with Boltzman equation, the obtained Vm were -42.56 ± 0.81 for WT, -41.11 ± 0.82 for p.R965C, and -34.87 ± 1.80 for p.1876insM (p < 0.01). Slope factors were 5.61 ± 0.71 for WT, 4.71 ± 0.77 for p.R965C and 8.09 ± 1.65 for p.1876insM (p < 0.05). Besides, both p.R965C and p.1876insM showed a left shift in the steady state inactivation curve when compared to WT, which suggested a reduced availability of open channel under physiological condition. The obtained inactivation Vm were -84.30 ± 1.20 for WT, -93.70 ± 0.84 for p.R965C (p < 0.01), and -92.80 ± 0.90 for p.1876insM (p < 0.05). Slope factors were 7.89 ± 1.06 for WT, 6.67 ± 0.73 for p.R965C and 8.33 ± 0.81 for p.1876insM.

### Time constants of fast inactivation

On stimulation, sodium channel open and inactivated rapidly. Fast inactivation was accessed by analysis of time constant of fast inactivation. By fitting the decaying phase of sodium current, we found that the time constants of inactivation for p.1876insM were significant larger than WT (Figure 3B). This suggested that p.1876insM inactivated slower than WT during depolarizing stimulation.

### Recovery from inactivation and development of slow inactivation

**Figure 4:**
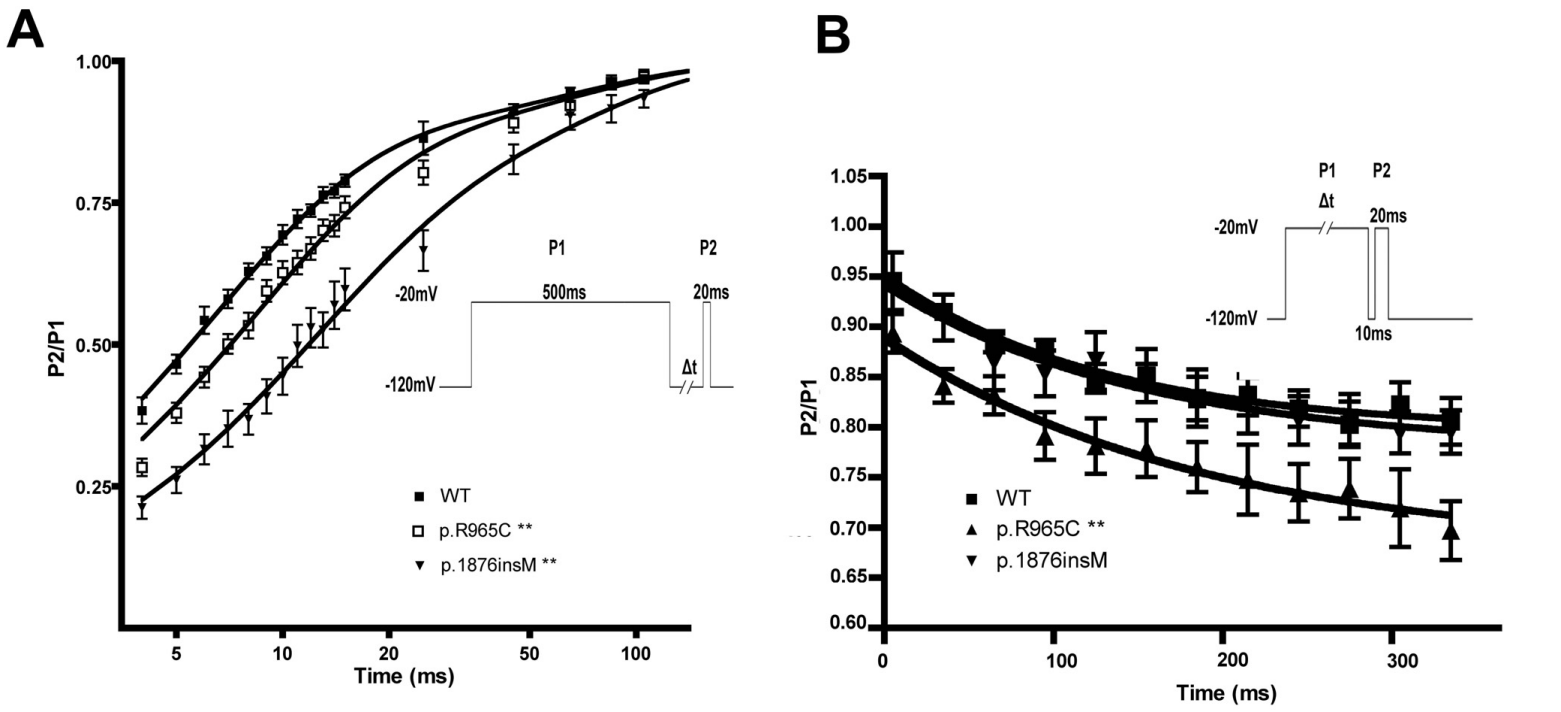
**Recovery from inactivation and develop of slow inactivation**. (A) Recovery from inactivation (n = 10,11, and 8 for WT, p.R965C, and p.1876insM respectively). The currents recorded at P2 were normalized to that at P1 and were plotted against time. Two-exponential equation was used to fit the plot. (B) Develop of slow inactivation. Currents at P2 and P1 were normalized and plotted against time (n = 8, 10, and 6 for WT, p.R965C, and p.1876insM respectively). **, p < 0.01 when compared to WT using one way ANOVA followed by Tukey test

Sodium channel inactivates rapidly by depolarizing stimuli but also recovers from inactivation rapidly during hyperpolarizing potential between depolarizing stimuli. Recovery from inactivation was evaluated by applying a long depolarizing pulse (20 mV, 500 ms) followed by a hyperpolarizing pulse (-120 mV) with time intervals to allow the channels to recover from inactivation. During the hyperpolarizing pulse, a brief depolarizing pulse was applied and the current elicited at this stage was analyzed. When the data were fitted with two exponential equation, the obtained parameters were as followed. For WT, A = 0.83 ± 0.03, *τ*f = 6.19 ± 0.33, and *τ*s = 59.85 ± 11.17. For p.R965C, A = 0.82 ± 0.15, *τ*f = 8.06 ± 0.61 (p < 0.01), and *τ*s = 66.12 ± 21.16. For p.1876insM, A = 0.65 ± 0.12, *τ*f = 10.7 ± 1.96 (p < 0.01), and *τ*s = 58.76 ± 19.85. This suggested that both mutants showed a significantly slower recovery from inactivation (Figure. 4A). Besides the fast inactivation, cardiac sodium channel might enter a more stable state of inactivation called slow inactivation upon prolonged depolarizing stimuli. Here we apply a depolarizing pulse (20 mV) with time intervals as in Figure 3B to access this property. We found that p.R965C channel was more prone to develop slow inactivation and this was statistically significant (Figure 4B).

### Cell surface protein biotinylation and Western blotting

**Figure 5:**
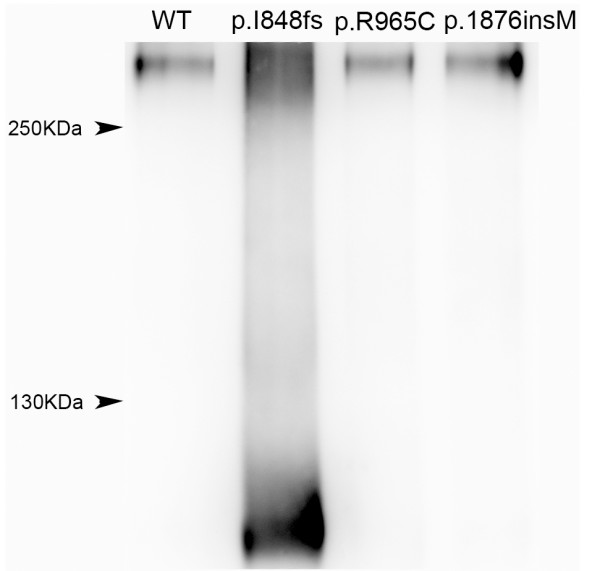
**Representative western blotting of WT, p.I848fs. p.R965C, and p.1876insM SCN5A channels from 4 independent experiments**. HEK293T transfected with WT or mutant plasmid were firstly treated with cell impermeable biotinylation reagent and total proteins were extracted from these cells. After immunoprecipitated with anti-myc antibody, which recognized the epitope added on all SCN5A constructs, the immunoprecipitated complex were subject to western blotting and reacted with HRP conjugated streptavidin. All four SCN5A constructs displayed bands by western blotting, indicating they were all subject to biotinylation reaction. This suggested that they all reached cytoplasmic membrane. Note that p.I848fs produced a truncated protein with a smaller molecular weigh than others.

SCN5A encodes the alpha subunit of cardiac sodium channel and therefore its functional destination in cells should be the cell surface membrane. We performed cell surface protein biotinylation reaction to access whether SCN5A protein reached cell surface membrane. In this reaction, only cell surface proteins were exposed to biotinylaing reagent and added biotins on them. After immunoprecipitated with anti-myc antibody, the immunoprecipatated complexes were subject to western blotting and analyzed by HRP conjugated streptavidin, which recognized biotinylated proteins. Clear presence of biotinylated proteins for all constructs (WT, p.I848fs, p.R965C, and p.1876insM) was observed (Figure 5). This suggested that they were all capable to reach to cell surface.

### Intracellular trafficking analysis by confocal imaging

**Figure 6:**
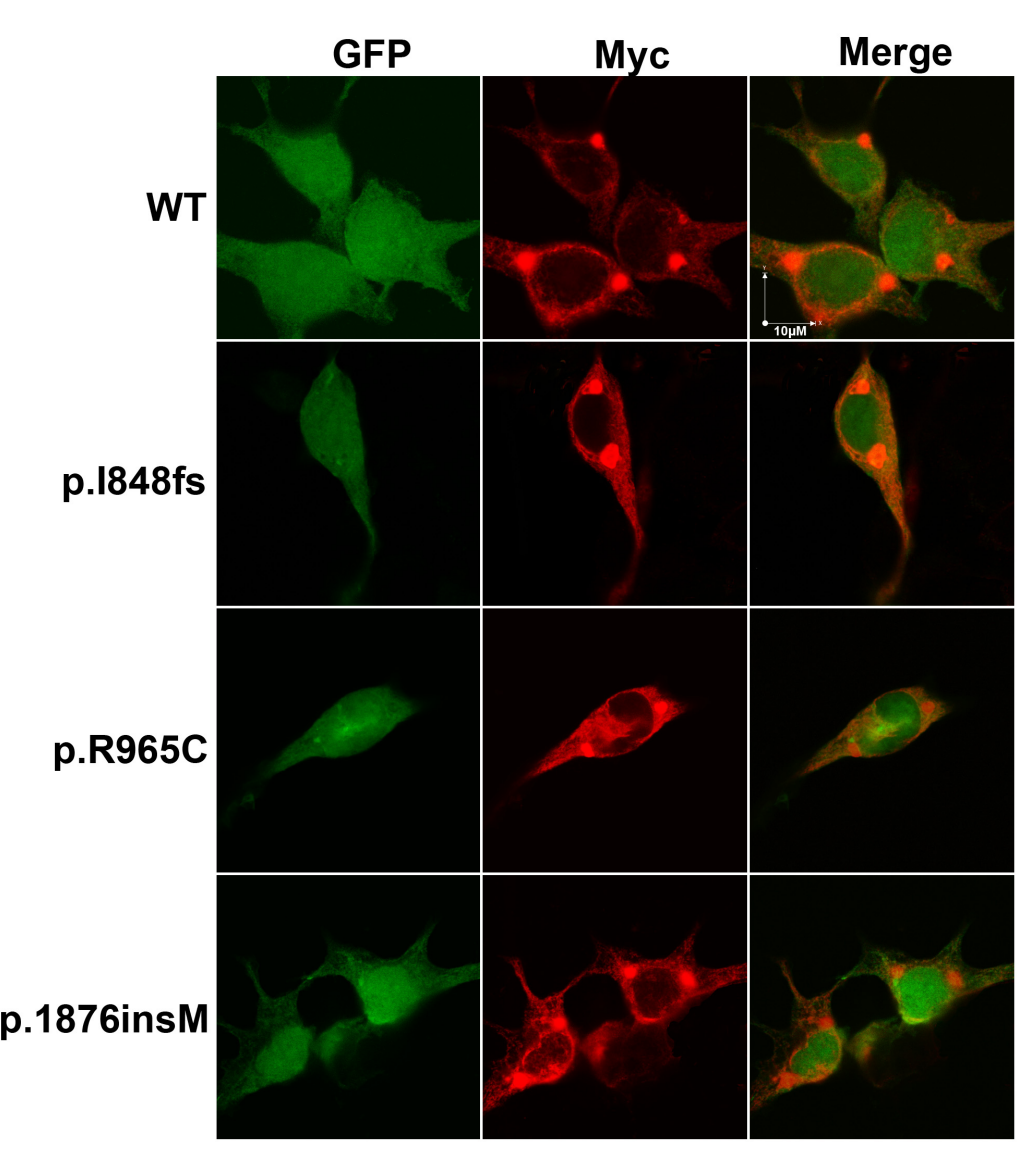
**Immunocytochemistry staining revealed by confocal image (total cells analyzed were 25, 18, 6, and 9 for WT, p.I848fs, p.R965C, and p.1876insM respectively from 7 independent experiments)**. The presence of red fluorescence (anti-myc antibody) at cytoplasmic membrane indicated that WT as well as other mutants were all trafficking competent. The red spot at the center of cell might be immature SCN5A protein in ER.

The construct used in this study had a myc epitope fused to the N-terminus of SCN5A protein so that we might regard antibody that recognized myc as the one that target SCN5A. The construct also generated GFP protein dicistronically and we might therefore regard the presence of GFP protein as the presence of transfected cells. Similar intracellular distribution for WT and mutants SCN5A proteins, and the presence of red florescence at the periphery of cells were observed. This suggested that p.I848fs as well as others were trafficking competent (Figure 6). There were some strong 'red spot', and this might due to the immature proteins in ER pool.

## Discussion

We characterized three mutations in SCN5A among Taiwanese patients with Brugada syndrome using patch clamp technique, western blotting and confocal imaging. Western blotting and confocal imaging showed that three mutants were trafficking-competent. However, their electrophysiological properties were impaired when compared with WT. p.I848fs elicited no sodium current. p.R965C produced sodium channel with impaired steady state inactivation, recovery from inactivation and slow inactivation while p.1876insM generated sodium channel with altered kinetics of activation, inactivation, and recovery from inactivation. We recognized that a decreased of sodium current might contribute to Brugada syndrome because all the three mutations resulted in a decrease of sodium current. However, the degree of sodium channel decrease does not seem to correlate with conduction time such as PR interval. Some other factors might compensate or play unknown roles in the clinical presentation.

SCN5A encodes the alpha subunit of cardiac sodium channel, which consists four homologous domains, and each domain contains six alpha-helical transmembrane repeats[3]. The frame shift of the p.I848fs mutant located at the fifth transmembrane segment of domain 2 (DII/S5) of cardiac sodium channel. Because it produces a protein with a truncation of more than a half of the WT cardiac sodium channel protein, the observation that it elicited no current was reasonable. In constancy with our founding, Shin et al. reported a mutation, W1119X, which lacked even less amino acids than p.I848fs, failed to generate any current[14]. Moreover, by western blotting and confocal imaging analysis, the presence of p.I848fs at cytoplasmic membrane suggested that the remained amino acid residues in I848fs were sufficient for the processing and trafficking of cardiac sodium channel.

The substituted amino acid p.R965C located at the intracellular loop between domain II and domain III of SCN5A protein (DII-DIII). Mutations located at this region had been reported , but few had been characterized by functional studies. Arginine at position 965, together with nearby amino acids, formed a specialized structure with putative amphiphilic helix carrying regularly arranged positive charges which resembling an S4 segment of voltage-gated ion channels[15]. The sequence here was unique to cardiac sodium channel. Camacho et al. had reported a splice variant lacking this sequence and showed that was associated with altered steady state activation, and inactivation[15]. Besides, Camacho et al. also pointed out that three positively charged arginine in this region were involved in current density. Arginine at 965 position located at one of them (the other two were at 968 and 971). The substitution of p.R965C from the positively charged aginine to a neutral cysteine might influence channel gating properties. In this study we observed a left shift in inactivation curve, and altered recovery from inactivation. The current density was not significantly changed, although it seemed to be smaller. Besides, the altered steady state inactivation might partly be due to impaired slow inactivation as we observed.

There had been many functional studies reported for the mutations in the carboxyl-terminal of cardiac sodium channel and implied its role in controlling channel inactivation [16-18]. As predicted by the amino acid segment, the carboxyl-terminal domain of cardiac sodium channel can to be divided into two parts. The proximal part was structured and forms six helices, whereas the distal part (about 100 amino acids) is unstructured. Loss of the predicted six helices greatly destabilizes inactivation while truncation of the unstructured part does not affect no channel gating[19]. The six helices of the carboxyl -terminal might interact with the linker of domain III and domain IV during inactivation. Changes of amino acid at these regions influences inactivation and contributes to Brugada syndrome, LQT3 or both, such as del KPQ1507-1509 (DIII-DIV), E1784K (C-terminal) or 1795insD(C-terminal) [20-23]]. The insertion of the p.1876insM mutant located at the fifth helices of the carboxyl-terminal and this mutation was found to have an altered inactivation. This was in agreement with the previous reports [16-18]. Besides inactivation, mutations located at the carboxyl-terminal might affect other gating parameters [20-22,24-26]. Rivolta et al. reported two mutations, Y1795H and Y1795C, contributing to Brugada syndrome and LQT3 respectively [24]. These two mutations affected steady state inactivation, fast inactivation, current density, and were more prone to enter slow inactivation without affecting steady state activation and recovery from inactivation. Similar findings were also observed in the Brugada syndrome mutation, C1859S as reported by Petitprez et al[25]. In this study, we found that the p.1876insM mutant at the carboxyl-terminal affected more than these parameters. Change of steady state activation of p.1876insM was observed as Shirai et al. observed in T1620M and S1710L [26]. This again proved the importance of carboxyl-terminal in regulation of cardiac sodium channel.

Some residues of cardiac sodium channel were important in regulation of the gating property of channel. Changes or lacks of these residues might therefore contribute to the alteration of electrophysiological property as we showed in this study. The three mutations accounted for Brugada syndrome all had electrophysiological alteration and contributed to loss of function of cardiac sodium channel. A more detailed screening of relationship between structure and channel gating might be worthwhile since similar results were observed for mutations located at different positions.

## Competing interests

The authors declare that they have no competing interests.

## Authors' contributions

C-HH carried out the molecular genetic studies, participated in the sequence alignment and drafted the manuscript. W-PC carried out the cellular electrophysiological studies. CTT carried out the immunoassays. H-MT participated in the sequence alignment. J-LL, Y-BL and J-MJ participated in the design of the study and performed the statistical analysis. M-JS and L-PL conceived of the study, and participated in its design and coordination. All authors read and approved the final manuscript.
